# The Surgical Treatment for Portal Hypertension: A Systematic Review and Meta-Analysis

**DOI:** 10.1155/2013/464053

**Published:** 2013-01-27

**Authors:** Lanning Yin, Haipeng Liu, Youcheng Zhang, Wen Rong

**Affiliations:** Department of General Surgery, Second Hospital of Lanzhou University, 82 Cuiyingmen, Chengguan District, Lanzhou, Gansu 730030, China

## Abstract

*Aim*. To compare the effectiveness of surgical procedures (selective or nonselective shunt, devascularization, and combined shunt and devascularization) in preventing recurrent variceal bleeding and other complications in patients with portal hypertension. *Methods*. A systematic literature search of the Medline and Cochrane Library databases was carried out, and a meta-analysis was conducted according to the guidelines of the Quality of Reporting Meta-Analyses (QUOROM) statement. *Results*. There were a significantly higher reduction in rebleeding, yet a significantly more common encephalopathy (*P* = 0.05) in patients who underwent the shunt procedure compared with patients who had only a devascularization procedure. Further, there were no significant differences in rebleeding, late mortality, and encephalopathy between selective versus non-selective shunt. Next, the decrease of portal vein pressure, portal vein diameter, and free portal pressure in patients who underwent combined treatment with shunt and devascularization was more pronounced compared with patients who were treated with devascularization alone (*P* < 0.05). *Conclusions*. This meta-analysis shows clinical advantages of combined shunt and devascularization over devascularization in the prevention of recurrent variceal bleeding and other complications in patients with portal hypertension.

## 1. Introduction

Portal hypertension substantially affects the patient quality of life and leads to high mortality. In developing countries, the incidence of portal hypertension is significantly higher than that in developed countries [1]. Recurrent variceal hemorrhage and hepatic failure are common causes of death in these patients. About one-third of patients with liver cirrhosis and varices experience hemorrhages [2]. The mortality due to first variceal bleeding can be as high as 30–50% [3]. The following treatment options are currently available for this disease.

Nonselective *β*-blockers and endoscopic variceal ligation have been utilized to prevent first variceal hemorrhage. A recent meta-analysis on nonselective *β*-blockers demonstrated that these drugs reduce rebleeding and increase survival [4]. However, only one-third of patients have hepatic venous pressure gradient (HVPG) response while on beta-blockers [5]. 

Currently, the safest method to treat acute bleeding from uncomplicated gastroesophageal varices is endoscopic sclerotherapy [6]. Unfortunately, this method does not reduce the bleeding risk in patients with accompanying liver cirrhosis [6]. Further, sclerotherapy is not effective for primary prevention of variceal bleeding [6]. In addition, esophagogastric variceal bleeding cannot be controlled, or relapses within 24 hours, in approximately 20% of patients with portal hypertension. 

Another treatment option is to decrease variceal pressure via portal systemic shunting or eradication of esophageal varices. This is most commonly achieved by shunt and devascularization. Beside prevention of variceal hemorrhage, both these procedures have the goal to maintain portal perfusion and preserve hepatocyte function [7]. Shunt and devascularization differ in their effects on the hemodynamics of the portal venous system [8]. The total hepatic blood flow is significantly lower in patients after shunt operation compared with patients after devascularization. The hepatic venous pressure gradient was also decreased in the shunt operated patients, in contrast to patients with devascularization operation. Distal splenorenal shunt has a significantly lower morbidity, compared with nonselective shunting procedures, but leads to similar incidence of portal-systemic encephalopathy, shunt occlusion, and recurrent hemorrhages [9, 10]. Devascularization includes splenectomy and devascularization of the upper half of the stomach and the lower third of esophagus; its beneficial effects are not enhanced by its combination with portal venous perfusion [11].

Given that there is no consensus over the best current surgical therapy for variceal bleeding in patients with portal hypertension, we conducted this study to evaluate the effects of selective shunt, nonselective shunt, devascularization, and combined shunt and devascularization, which were the most commonly used procedures on variceal hemorrhage, encephalopathy, ascites, mortality, and improvement of postoperative hemodynamics.

## 2. Methods

### 2.1. Inclusion and Exclusion Criteria

To ensure the high quality of this meta-analysis of Randomized Controlled Trials (RCTs), trial selection, data analyses, and presentation of results were carried out according to the guidelines of the Preferred Reporting Items for Systematic Reviews and Meta-Analyses (PRISMA) statement [12]. Clinical studies included randomized controlled trials (RCTs) with or without blinding. The study individuals were patients with portal hypertension, without any limitation on nationality or ethnicity. The following interventions were compared: devascularization versus shunt, devascularization versus combined shunt and devascularization, and selective shunt versus nonselective shunt. Study outcomes were rebleeding, portosystemic encephalopathy, ascites, mortality, systemic hemodynamic evaluation (including portal vein pressure), portal vein diameter, and free portal pressure. Exclusion criteria were the following: (1) RCTs on etiology, mechanism, diagnosis, or prevention of portal hypertension; (2) interventions: sclerotherapy, drug therapy, any surgical treatment other than shunt, devascularization, or combined shunt and devascularization.

### 2.2. Outcomes

The primary study outcomes were rebleeding (defined as cases with a history of variceal bleeding before surgery, or variceal bleeding during the followup after surgical treatment), encephalopathy, and ascites. The secondary outcomes were late mortality, long-term hemodynamic changes (including free portal pressure, andd portal vein pressure, portal vein diameter), and portal hypertensive gastropathy.

### 2.3. Search Strategy

The following electronic databases were searched in October 2010 to retrieve studies for potential inclusion: Cochrane Central Register of Controlled Trials (CENTRAL), MEDLINE (via PubMed), and EMBASE. We also searched Chinese academic journals, such as CNKI, VIP, Wan Fang, and CBM. These databases represent the most frequently searched databases for medical systematic reviews. We also hand searched the Chinese Journal of Surgery (1972–1992). The search terms were “portal hypertension,” “devascularization,” and “shunt.” Reference lists of retrieved relevant publications were also searched for additional trials.

### 2.4. Validity Assessment and Data Collection

Each of the retrieved publications was independently assessed by two authors. All relevant data from each publication, including study design, patient numbers, length of the followup, principal findings, and conclusions, were collected. Whenever feasible, meta-analyses were carried out on all available parameters, which required that any specific parameter was to be addressed by at least two RCTs and that adequate data were provided for statistical analysis. When a parameter was analyzed only by one publication, we resorted to describing this parameter.

### 2.5. Assessment of Risk of Bias

The characteristics of our study and the quality of each included RCTs were evaluated using the Cochrane Collaborations tool for assessing risk of bias [13]. Briefly, the assessment criteria were applied to the following principal domains: (1) generation sequence and concealment of allocation; (2) blinding of caregivers, participants and outcome assessors; (3) incomplete outcomes; and (4) selective reporting. Studies which were deemed as “adequate” in all principal domains were considered to be of low risk of bias. Studies, in which there was no clear judgment concerning the procedures in one or more key domains, were considered to be at least of medium risk of bias. Studies with clearly inadequate procedures in one or more of the key domains were considered to be of high risk of bias. In this context, blinding of operators was impossible and blinding of patients meaningless. However, RCTs were evaluated as medium risk of bias when blinding of participants and outcome assessors, concealment of allocation, or randomization method were unclear. For each RCT, the risk of bias was independently assessed by two authors. The summary assessments of risk of bias were evaluated not only for each RCT across outcomes but also specifically for meta-analyses. RCTs with unclear or high risk of bias were not excluded from meta-analyses. However, meta-analyses including such RCTs were explicitly indicated. As mentioned above, nonrandomized prospective trials and nonrandomized retrospective analyses were excluded from meta-analysis.

### 2.6. Statistical Analysis

Statistical analysis was done using Review Manager (RevMan) software version 5.0 (The Nordic Cochrane Centre, The Cochrane Collaboration, Copenhagen, Denmark). Statistical heterogeneity was tested using chi-square and *I* ^2^ tests. Data were pooled using a fixed-effect model if heterogeneity was limited; a random-effect model was used when there was a significant heterogeneity among the trials. The conventional 0.05 level of significance was employed. The RCTs were analyzed separately from nonrandomized studies. Results are expressed as mean ±  SE and were analyzed by paired and unpaired Student's *t*-test.

## 3. Results

### 3.1. Identification of Eligible Clinical Trials

**Figure 1:**
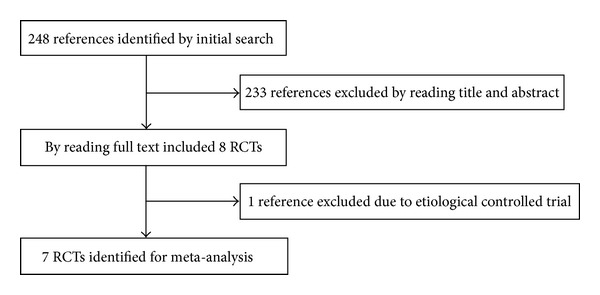
Flow chart showing how trials were identified for inclusion in review.

The search strategy generated 272 citations from English databases. In total, we found 16 RCTs comparing shunt with devascularization, selective with nonselective shunt, or shunt or devascularization with combined therapy (Figure 1, Table 1) [14–30]. Among these RCTs, there were 4 trials comparing shunt with devascularization, 5 on selective versus nonselective shunt, 4 on shunt or devascularization versus combined treatment, and 3 trials on other procedures.

### 3.2. Patient Characteristics

From 16 RCTs, 1042 patients were included in the meta-analysis. Patients admitted to hospital between 1970 and 2010 with recent history of variceal bleeding were considered eligible for inclusion in the meta-analysis if they met the following criteria: (1) recent episode of bleeding from esophageal varices assessed by emergency endoscopy which showed spurting or oozing varices; a previously placed endoscopic band or a clot on the varices; esophageal varices without any other lesion in the stomach or duodenum, (2) a bleeding stop for at least 2 weeks before recurring, (3) superior mesenteric, splenic, and portal veins were on angiogram, and (4) no history of intractable ascites or chronic encephalopathy. Patients were not included in the meta-analysis if they had any of the following: (1) serum bilirubin concentration above 50 mmol/L, prothrombin rate below 45%, serum aspartate aminotransferase above 3N, or serum alpha-fetoprotein concentration above 20 ng/L, (2) evidence of hepatocellular carcinoma, (3) comorbidities reducing life expectancy (e.g., ongoing cancer), or (4) inability to undergo regular surveillance. 

The patients' data are presented in Table 2. There were no significant differences among patients included in the meta-analysis.

### 3.3. Characteristics of Included RCTs

The randomization method was described in three RCTs. These RCTs attempted no blinding or allocation concealment. Thirteen RCTs reported loss of followup, while 14 RCTs reported the time of followup (Table 1).

### 3.4. Rebleeding

**Figure 2:**
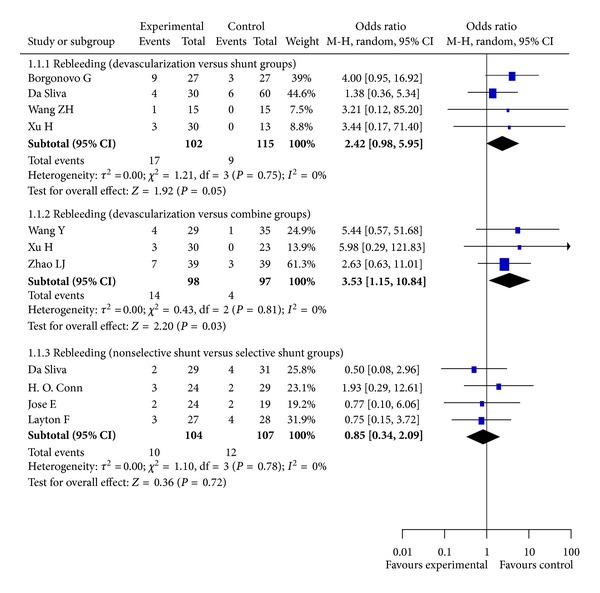
Meta-analysis of devascularization and shunt groups, devascularization and combined groups, and nonselective shunt and selective shunt groups in RCTs.

Rebleeding was reported in 10 RCTs. Our meta-analysis shows that the rate of rebleeding in the devascularization group was significantly higher than in patients with shunt (*P* = 0.05, Odds Ratio M-H, Random, 95%  CI = 2.42 [0.98, 5.95], *I* ^2^ = 0%; Figure 2) or combined therapy (*P* = 0.03, Odds Ratio M-H, Random, 95%  CI = 3.53 [1.15, 10.84], *I* ^2^ = 0%; Figure 2). There was no statistical difference between patients undergoing selective or nonselective shunt (*P* = 0.72, Odds Ratio M-H, Random, 95%  CI = 0.85 [0.34, 2.09], *I* ^2^ = 0%; Figure 2).

### 3.5. Encephalopathy

**Figure 3:**
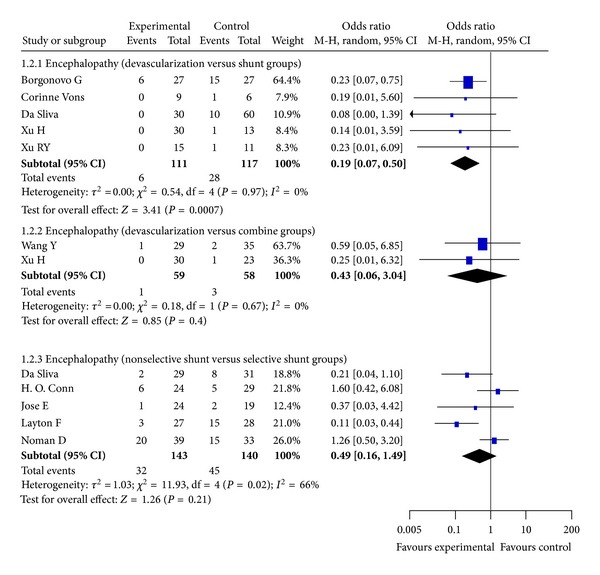
Meta-analysis of devascularization and shunt groups, devascularization and combined groups, and nonselective eshunt and selective shunt groups in RCTs.

Encephalopathy was reported in 11 RCTs. Our meta-analysis shows that the rate of encephalopathy in the shunt group was significantly higher compared with the devascularization (*P* = 0.0007, Odds Ratio M-H, Random, 95%  CI = 0.19 [0.07, 0.50], *I* ^2^ = 0%; Figure 3), but not in the combined treatment group (*P* = 0.4, Odds Ratio M-H, Random, 95%  CI = 0.43 [0.06, 3.04], *I* ^2^ = 0%; Figure 3). The comparison between selective and nonselective shunt groups revealed no significant differences (*P* = 0.21, Odds Ratio M-H, Random, 95%  CI = 0.49 [0.16, 1.49], *I* ^2^ = 66%; Figure 3).

### 3.6. Ascites

**Figure 4:**
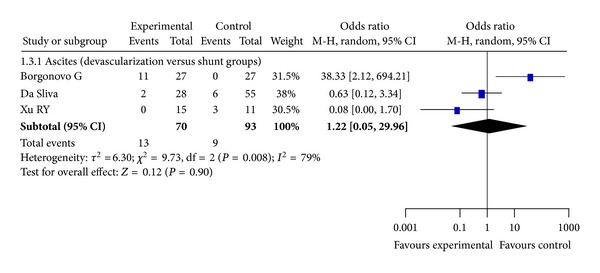
Meta-analysis of devascularization and shunt groups in RCTs.

Ascites was reported in 3 RCTs. Our meta-analysis demonstrates that the ascites rates were not significantly different between the devascularization and shunt groups (*P* = 0.90, Odds Ratio M-H, Random, 95%  CI = 1.22 [0.05, 29.96], *I* ^2^ = 79%; Figure 4).

### 3.7. Late Mortality

**Figure 5:**
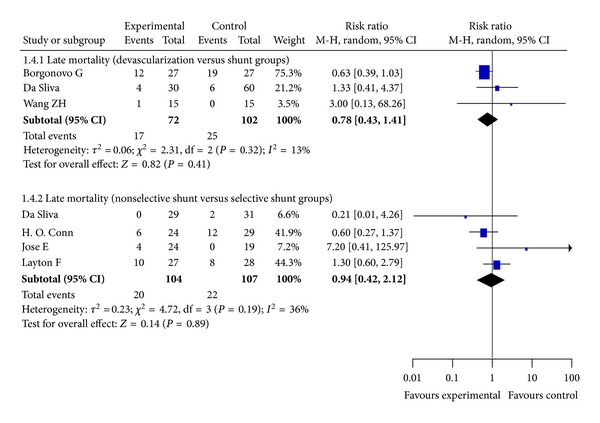
Meta-analysis of devascularization and shunt groups, nonselective shunt and selective shunt groups in RCTs.

Late mortality (i.e., after ten years) was reported in 7 RCTs. Our meta-analysis shows that the rate of late mortality was not significantly different between the devascularization and shunt groups (*P* = 0.41, Odds Ratio M-H, Random, 95%  CI = 0.78 [0.43, 1.41], *I* ^2^ = 13%;Figure 5) or the selective versus nonselective shunt groups (*P* = 0.89, Odds Ratio M-H, Random, 95%  CI, *I* ^2^ = 36%; Figure 5).

### 3.8. Hemodynamics

**Figure 6:**
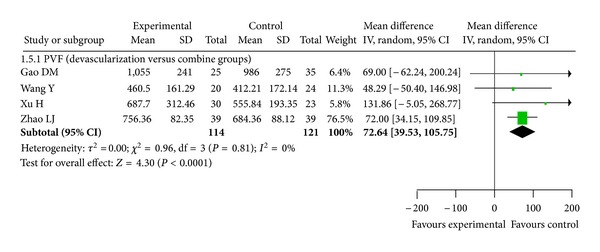
Meta-analysis of devascularization and shunt groups for portal vein pressure.

**Figure 7:**
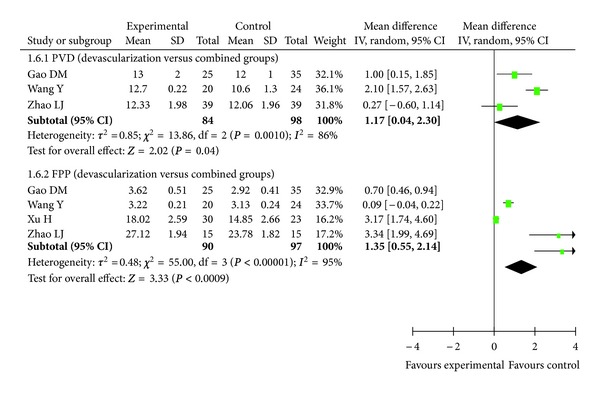
Meta-analysis of devascularization and shunt groups, devascularization and combined groups for portal vein diameter and free portal pressure.

Because of the high incidence of hepatitis B and schistosomiasis, the rates of portal hypertension in China are significantly higher than in developed countries [1]. We could not find any article on hemodynamics from researchers outside China; therefore, we resorted to analyzing publications by Chinese authors. The following RCTs [18–21] demonstrated a significant decrease of free portal pressure in the combined treatment group compared with devascularization alone (*P* < 0.05 for all comparisons; data not shown). These RCTs further indicated a significant decrease of portal vein diameter and portal vein pressure in both combined treatment and devascularization groups (*P* < 0.05 for all comparisons; data not shown). In one RCT [21], there was a significant postoperative decrease of free portal pressure and portal vein pressure in the shunt group compared with the devascularization group (*P* < 0.05 for all comparisons; data not shown). Another RCT [26] showed a significant decrease of portal vein diameter in both the combined treatment and devascularization groups (*P* < 0.001; data not shown). The results of portal vein pressure are shown in Figure 6 (*P* < 0.0001, Odds Ratio M-H, Random, 95%  CI = 72.64 [39.53, 105.75], *I* ^2^ = 0%), while the results of portal vein diameter and free portal pressure are presented in Figure 7 (*P* = 0.04, Odds Ratio M-H, Random, 95%  CI = 1.17 [0.04, 2.30], *I* ^2^ = 86%, and *P* = 0.0009, Odds Ratio M-H, Random, 95%  CI = 1.35 [0.55, 2.14], *I* ^2^ = 95%). Our meta-analysis indicates a significant decrease of portal vein pressure, portal vein diameter, and free portal pressure in the combined treatment group compared with the devascularization group (*P* < 0.0001).

### 3.9. Portal Hypertensive Gastropathy

One RCT [15] described effects of splenectomy in combination with ligation of the left gastric vein (coronary vein) and shunt on portal hypertensive gastropathy. Specifically, splenectomy in combination with ligation of the left gastric (coronary) vein exacerbates portal hypertensive gastropathy (*P* < 0.05; data not shown), while shunt reverses this (*P* < 0.05; data not shown).

## 4. Discussion

Surgical treatment remains the best treatment choice for patients with portal hypertension to prevent life-threatening bleedings. Indeed, surgery has a lower rebleeding rate compared with other forms of treatment [31, 32]. However, no single surgical treatment has been recognized as an ideal approach for all cases of portal hypertension with variceal bleeding. The choice of surgical treatment for these patients must balance out the risks of recurrent bleeding, encephalopathy, and hepatic failure [33]. In the RCTs included in our meta-analysis, there were 309 patients who underwent esophagogastric devascularization and splenectomy and 29 patients who underwent the Hassab procedure (splenectomy and devascularization operation). Shunts included selective and nonselective shunts: 244 patients received selective shunts, 232 nonselective shunts, and 217 received a combined treatment. We observed no significant differences in rebleeding, late mortality, and encephalopathy between selective and nonselective shunts. By contrast, there was a significant reduction in the rate of rebleeding in patients who underwent any shunt procedure compared with those who had a devascularization procedure. Recurrent hemorrhages in patients with distal splenorenal shunts were commonly associated with shunt occlusion. Variceal hemorrhage and portal hypertensive gastropathy were the two sources of rebleeding in portal hypertension. It was reported that shunt procedure has a positive effect on portal hypertensive gastropathy [15]. Our meta-analysis further demonstrates a significant reduction in the rate of encephalopathy in patients who underwent devascularization procedure compared with those with a shunt procedure. Several RCTs showed that the rate of encephalopathy in the shunt group was higher than that in the devascularization or combined treatment groups. This phenomenon may be related to splenectomy with gastroesophageal devascularization directly interrupting the extramural gastroesophageal collateral blood flow to the varices, while a distal splenorenal shunt creates a low-pressure drainage pathway by diverting the short gastric venous flow to the renal vein via the splenic venous system. Therefore, a distal splenorenal shunt seems to decompress, while splenectomy with gastroesophageal devascularization decongests the variceal channels [7]. Since there is a shunt from the portal vein to systemic venous circulation, the incidence of hepatic encephalopathy is expected to be increased.

Our meta-analysis further shows that the rates of late mortality and ascites were not significantly different between study groups; that is, the incidences of clinically apparent ascites were similar among survivors of all procedures. Ascites occur late after operation and should probably be considered part of the natural history of portal hypertension and chronic liver disease. The rate of long-term survival reflects the deleterious effects of the progressive cirrhotic process on the intrahepatic vascular system, functional hepatic reserve, and hepatocyte failure after surgical procedures. The goal of the treatment of portal hypertension caused by cirrhosis is not only the maintenance of hepatic function, but also a decrease in the portal pressure and elimination of feeding vessels to the varices. Four RCTs reported combined therapy consisting of splenectomy, splenorenal shunt, and esophagogastric devascularization. Regarding hemodynamics, our meta-analysis shows a significant decrease of portal vein pressure, portal vein diameter, and free portal pressure in the combined treatment group compared with the devascularization group. Combined procedures integrate the advantages of shunt with those of devascularization, including maintaining the normal anatomic structure of the portal vein. Combined procedures should, therefore, be considered as one of the best choices for surgical intervention in inpatients with portal hypertension. A number of surgical procedures have been developed to manage esophageal varices [34]. Inokuchi et al. [3] stated that variceal hemorrhage was the most frequent complication. Still, surgery should not be used for primary prophylaxis [3]. Endoscopic sclerotherapy and ligation are commonly used to treat esophageal varices [35, 36]. Endoscopic treatments are less invasive than surgery but have poorer long-term results [37].

Each meta-analysis holds shortcomings and biases [38–40]. First, meta-analyses may fail to identify significant differences if the sample sizes remain too small. Second, the quality of meta-analysis depends greatly on the quality of RCTs included. Therefore, we explicitly indicated the RCTs with low risk of bias providing the reader with the best available information for interpretation of the data. Moreover, to better address the heterogeneity of the available RCTs, assessments of each publication and extractions of relevant data were independently carried out by two authors. Considering the limited number of RCTs and the small number of patients included, we also used the random-effects model for all meta-analyses with respect to heterogeneous populations (Table 3). Because of the high incidence of hepatitis B and schistosomiasis in China, the incidence of portal hypertension is significantly higher than in developed countries [1]. A combination of shunt and devascularization was reported only in China, and there are six RCTs included in our meta-analyses that were designed in China, so most patients in our study are of Asian ethnicity. There were several RCTs conducted in Japan and other countries without full text provided, so we have excluded these articles.

In summary, our meta-analysis evaluated the incidence of variceal hemorrhage, encephalopathy, ascites, mortality, and postoperative systemic hemodynamic effects in four different surgical procedures: selective or nonselective shunt, devascularization, and combined shunt with devascularization. We conclude that the procedure of combined shunt and devascularization is the most suitable in prevention of recurrent variceal bleeding and other complications in patients with portal hypertension.

## Figures and Tables

**Table 1:** Summary of included RCTs.

Study	Year	Country	Sample size	Follow-up time	Outcomes
Wang et al. [14]	2002	China	30	1 year	PVF, FPP, HTF, R15 ICG
Xu et al. [15]	1997	China	26	Unclear	PHG
Vons et al. [16]	1996	France	15	6 months	Long-term hemodynamics
Borgonovo et al. [17]	1996	Italy	54	Unclear	Encephalopathy, rebleeding, survival, ascites, hepatocellular carcinoma
Xu et al. [18]	2003	China	66	7 years	Hemodynamics
Zhao et al. [19]	2009	China	78	6 months–5 years	Hemodynamics
Gao et al. [20]	2002	China	220	1–5 years	Hemodynamics
Wang et al. [21]	2000	China	64	6 months–20 years	Hemodynamics
de Cleva et al. [22]	2007	Brazil	36	6–84 months	Hemodynamics
Strauss et al. [23]	1999	Brazil	73	5–10 years	Size of gastroesophageal varices
da Silva et al. [24–26]	1986	France	94	>5 years	Encephalopathy, rebleeding, failure, mortality
Grace et al. [27]	1988	Boston	81	Mean of 3.5 years	Late mortality, cumulative survival, hemorrhage from varices, encephalopathy
Warren and whithead [28]	1986	USA	55	11 years	Rebleeding, hepatic cell function, quality of life
Conn et al. [10]	1981	USA	53	54 months	Encephalopathy, rebleeding, mortality
Fischer et al. [29]	1981	USA	42	60 months	Encephalopathy, rebleeding, mortality
Orozco et al. [30]	1994	USA	55	>16 months	Encephalopathy, mortality, hepatic portal perfusion, hepatic function

PVF: portal vein pressure; FPP: free portal pressure; PHG: portal hypertensive gastropathy; HTF: total hepatic flow; R15: ICG indocyanine green retention rate at 15 min.

**Table 2:** Baseline characteristics of 1042 patients in 16 RCTs.

Study	Age	Male : female ratio	Child-Pugh's classification	Cirrhosis	History of gastrointestinal bleeding	Lost to followup
Wang et al. [14]	20–57	23 : 7	A, B	30/30	Yes	0/30
Xu et al. [15]	47.5 (30–68)	22 : 4	A, B	26/26	Yes	0/26
Vons et al. [16]	51 ± 8	10 : 5	Unclear	15/15	Yes	0/15
Borgonovo et al. [17]	52.3 ± 9.35	43 : 11	A, B	54/54	Yes	0/54
Xu et al. [18]	21–59	35 : 31	A (19) B (35) C (12)	66/66	Unclear	0/66
Zhao et al. [19]	30–71	54 : 24	A (44) B (34)	Unclear	Yes	0/78
Gao et al. [20]	43.2	Unclear	A (59) B (162) C (7)	Unclear	Yes	36/220
Wang et al. [21]	45.2	43 : 21	A (46) B (18)	Unclear	Yes	0/64
de Cleva et al. [22]	22–56 (range 39)	1 : 1	Unclear	Unclear	Yes	0/36
Strauss et al. [23]	18–55	Unclear	Unclear	Unclear	Yes	Unclear
da Silva et al. [24–26]	18–55	63 : 31	Unclear	Unclear	Yes	Yes
Grace et al. [27]	53	68 : 13	A (43) B (35) C (3)	81/81	Yes	Unclear
Warren and whithead [28]	Unclear	Unclear	Unclear	55/55	Yes	0/55
Conn et al. [10]	50.7 ± 9	43 : 10	A (50%) B (40%) C (10%)	47/53	Yes	0/53
Fischer et al. [29]	32–69	Unclear	A (22) B (16) C (4)	36/42	Yes	Unclear
Orozco et al. [30]	50.1	35 : 20	Unclear	55/55	Yes	0/55

**Table 3:** Summary of risk of bias assessments of 16 RCTs.

Study	Randomized	Randomization method	Blinding	Allocation concealment	Intervention
Wang et al. [14]	Yes	Unclear	Unclear	Unclear	Shunt (15) versus devascularization (15)
Xu et al. [15]	Yes	Unclear	Unclear	Unclear	Shunt (11) versus devascularization (15)
Vons et al. [16]	Yes	Unclear	Unclear	Unclear	Portocaval shunt (6) versus Sugiura procedure (9)
Borgonovo et al. [17]	Yes	Unclear	Unclear	Unclear	Nonselective shunt (27) versus modified Sugiura procedure (27)
Xu et al. [18]	Yes	Unclear	Unclear	Unclear	SRS (13) versus PCDV (30) versus SRS + PCDV (23)
Zhao et al. [19]	Yes	Unclear	Unclear	Unclear	Sugiura procedure (39) versus SRS + PCDV
Gao et al. [20]	Yes	Table of random numbers	Unclear	Unclear	PCDV (100) versus SRS + PCDV (120)
Wang et al. [21]	Yes	Unclear	Unclear	Unclear	Devascularization + shunt (35) versus devascularization (29)
de Cleva et al. [22]	Yes	Unclear	Unclear	Unclear	EGDS (17) versus DSRS (19)
Strauss et al. [23]	Yes	Unclear	Unclear	Unclear	EGDS (25) versus DSRS (24) versus PSRS (24)
da Silva et al. [24–26]	Yes	Table of random numbers	Unclear	Unclear	EGDS (32) versus DSRS (30) versus PSRS (32)
Grace et al. [27]	Yes	Unclear	Unclear	Unclear	PSS (38) versus DSRS (43)
Warren and whitehead [28]	Yes	Unclear	Unclear	Unclear	Nonselective (29) versus selective shunt (26)
Conn et al. [10]	Yes	Unclear	Unclear	Unclear	DSRS (24) versus PSS (29)
Fischer et al. [29]	Yes	Card drawing	Unclear	Unclear	Selective (23) versus nonselective shunt (19)
Orozco et al. [30]	Yes	Unclear	Unclear	Unclear	Selective (27) versus nonselective shunt (28)

SRS: splenorenal shunt; PCDV: peripheral cardia divided vessel (division of left gastric vein/coronary vein); EGDS: esophagogastric devascularization and splenectomy; DSRS (DSS): distal splenorenal shunt; PSRS: proximal splenorenal shunt; PSS: portal-systemic shunt.
